# Phylogenetically widespread alternative splicing at unusual GYNGYN donors

**DOI:** 10.1186/gb-2006-7-7-r65

**Published:** 2006-07-25

**Authors:** Michael Hiller, Klaus Huse, Karol Szafranski, Philip Rosenstiel, Stefan Schreiber, Rolf Backofen, Matthias Platzer

**Affiliations:** 1 Institute of Computer Science, Chair for Bioinformatics, Albert-Ludwigs-University Freiburg, Georges-Koehler-Allee 106, 79110 Freiburg, Germany; 2 Genome Analysis, Leibniz Institute for Age Research - Fritz Lipmann Institute, Beutenbergstr. 11, 07745 Jena, Germany; 3 Institute of Clinical Molecular Biology, Christian-Albrechts-University Kiel, Schittenhelmstr. 12, 24105 Kiel, Germany; 4 Max Planck Institute for Molecular Genetics, Ihnestr. 63, 14195 Berlin, Germany

## Abstract

**Background:**

Splice donor sites have a highly conserved GT or GC dinucleotide and an extended intronic consensus sequence GTRAGT that reflects the sequence complementarity to the U1 snRNA. Here, we focus on unusual donor sites with the motif GYNGYN (Y stands for C or T; N stands for A, C, G, or T).

**Results:**

While only one GY functions as a splice donor for the majority of these splice sites in human, we provide computational and experimental evidence that 110 (1.3%) allow alternative splicing at both GY donors. The resulting splice forms differ in only three nucleotides, which results mostly in the insertion/deletion of one amino acid. However, we also report the insertion of a stop codon in four cases. Investigating what distinguishes alternatively from not alternatively spliced GYNGYN donors, we found differences in the binding to U1 snRNA, a strong correlation between U1 snRNA binding strength and the preferred donor, over-represented sequence motifs in the adjacent introns, and a higher conservation of the exonic and intronic flanks between human and mouse. Extending our genome-wide analysis to seven other eukaryotic species, we found alternatively spliced GYNGYN donors in all species from mouse to *Caenorhabditis elegans *and even in *Arabidopsis thaliana*. Experimental verification of a conserved GTAGTT donor of the *STAT3 *gene in human and mouse reveals a remarkably similar ratio of alternatively spliced transcripts in both species.

**Conclusion:**

In contrast to alternative splicing in general, GYNGYN donors in addition to NAGNAG acceptors enable subtle protein variations.

## Abstract 2

Computational and experimental evidence is given for alternative splicing at the unusual GYNGYN motif in several species, enabling in most cases subtle protein variations.

## Background

Given the rather limited number of human genes [1], alternative splicing is believed to be a major mechanism to bridge the gap between the gene and protein number [2,3]. Most human multi-exon genes express more than one splice variant [4]. Protein isoforms, produced by alternative splicing, can differ in various aspects, including ligand binding affinity, signaling activity, protein domain composition, subcellular localization, and protein half-life [5]. In coordination with nonsense-mediated mRNA decay, alternatively spliced transcripts can be degraded rapidly, providing a regulation and fine-tuning mechanism of the adjustment of the protein level [6].

The skipping of an exon is the most frequent alternative splice event, followed by alternative splice donor and acceptor sites [7]. Such splice events often result in large effects for the proteins, for example, by deleting functional units like protein domains [8,9] or transmembrane helices [10,11]. On the other hand, alternative splicing also allows the production of many very similar protein isoforms. The most frequent of these subtle events is the alternative splicing at NAGNAG or tandem acceptors [12]. In the NAGNAG motif (N stands for A, C, G or T/U; throughout the paper we write T instead of U also when referring to an RNA sequence), we have termed the upstream acceptor the E acceptor (since the downstream NAG becomes exonic in case of splicing at this site) and the downstream one the I acceptor (since the whole tandem becomes intronic). This splice acceptor motif frequently allows the selection of one of the two AGs in the splice process, resulting in the insertion/deletion (indel) of the I acceptor NAG in mRNAs, preferably if both Ns are either A, C, or T [13-15]. Despite the rather simple genomic structure, these NAG indels lead to a surprisingly high diversity at the protein level. Depending on the sequence of the up- and downstream exon and the phase of the intron, eight different single amino acid indels, the exchange of a dipeptide for an unrelated amino acid, and the indel of a stop codon are possible [12]. These subtle protein changes can result in functional differences for the respective protein isoforms [15-18].

The recognition of donor and acceptor splice sites is different. While the acceptor AG and its preceding polypyrimidine tract is recognized by the U2AF heterodimer [19], the donor site has an extended consensus sequence AG|GTRAGT (| is the splice site, R stands for A or G), that is bound by base pairing to the 5' end of the U1 snRNA [20]. However, two donor sites that are only three nucleotides (nt) apart would result in overlapping U1 snRNA binding sites and the GTNGTN motif differs from the donor consensus sequence at the two conserved positions +4 and +5. According to the consensus, an alternative usage of the GT dinucleotide 4 nucleotides downstream is much more likely but results in a frameshift and thus a dramatic change of the protein if the donor is located in the coding sequence (CDS).

Here we investigate whether alternative splicing at a GT or GC donor dinucleotide 3nt up- or downstream is possible. This type of alternative splicing requires a GYNGYN donor motif (Y stands for C or T) and is of interest because it would result in similar subtle protein changes like at NAGNAG tandem acceptors and thus increase the proteome plasticity. We found expressed sequence tag (EST) and/or mRNA evidence for alternative splicing at 110 human GYNGYN tandem donors and confirm the existence of both splice forms by RT-PCR experiments in seven cases. We report the occurrence of alternative splicing at GYNGYN tandem donors in six other animals and a plant. Analyzing the GYNGYN motifs that do and do not allow alternative splicing, we found significant differences in the stability of the U1 snRNA binding, conserved exonic and intronic flanks between human and mouse, and over-represented sequence motifs in the intronic flanks.

## Results

### Alternative splicing at tandem donor sites

**Figure 1:**
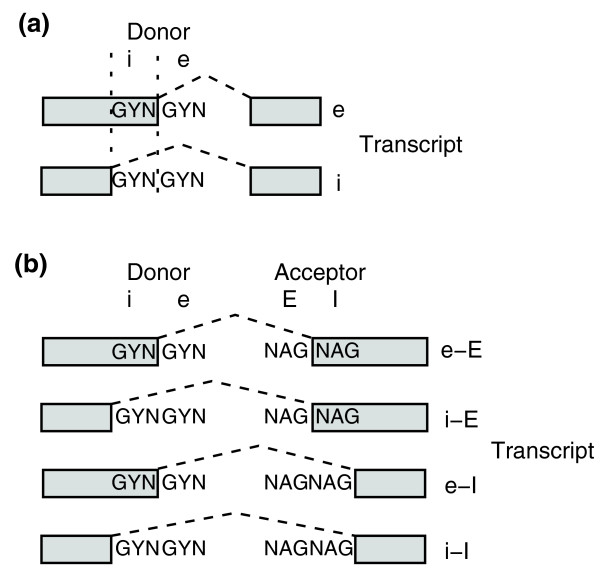
Nomenclature for tandem donor sites and transcripts. **(a) **Splicing at the downstream e donor makes the upstream GYN exonic while splicing at the upstream i donor makes the complete GYNGYN motif intronic. **(b) **Simultaneous usage of e or i donor and E or I acceptor results in four different transcripts (e-E, i-E, e-I, and i-I).

Although the great majority of introns begins with a GT dinucleotide, a small fraction of 0.76% begins with GC [1]. To investigate whether splice donor sites with the pattern GYNGYN allow the usage of both potential splice sites in humans, we first retrieved from the UCSC Human Genome Browser (hg17, May 2004) all RefSeq-to-genome alignments. Given the exon-intron structure of those transcripts, we extracted a 9 nucleotide sequence (3 exonic and 6 intronic nt; -3 to +6, no position 0) for all donor sites and checked the presence of a GYNGYN pattern. In agreement with the donor consensus sequence that shows no GY dinucleotide 3 nucleotides up- or downstream of the donor site, we found only 8,550 (5.2%) tandem donors from the total of 165,295 annotated donor sites (Table 1). Divided into the four different GYNGYN patterns, GTNGTNs and GCNGTNs are the most frequent ones while GCNGCN is very rare. Consistent with the proposed nomenclature for NAGNAG acceptors, we termed the upstream donor that renders the complete GYNGYN motif to be intronic the 'i donor'. Likewise, the other donor is called the 'e donor' because the upstream GYN becomes exonic using this donor (Figure 1a). Note that, inversely to NAGNAG acceptors, the 'e donor' is located downstream of the 'i donor'. We use lower case letters to denote the two donor sites and upper case letters for the two acceptor sites to distinguish between the transcripts that arise by alternative splicing at tandem donors or acceptors and between combinations of alternative donor and acceptor usage (Figure 1b; see also Discussion).

By searching dbEST and the human mRNAs from GenBank, we identified experimental evidence for alternative splicing at 110 (1.3% of 8,550) tandem donors (in the following we term these tandem donors 'confirmed') (Table 1; Additional data file 1). We term the remaining 8,440 donors 'unconfirmed' with the notion that they are enriched in GYNGYN donors that are not functional. The percentage of confirmed tandem donors is considerably higher for GTNGTN (2%) and GTNGCN (1.6%) patterns. No confirmed GCNGCN donor was found, presumably because this motif is very rare and because the weaker GC donor requires a more stringent sequence context. Since ESTs are random high-throughput samples from the transcriptome, spurious or mis-spliced entries may pollute dbEST, especially if the EST number for a particular locus is high [21,22]. However, the likelihood of splicing errors decreases if the respective splice event is represented by more than one EST and/or if the EST ratio between alternative splice forms is not extreme. From the 110 confirmed tandems, 50 (45%) have at least two ESTs and 19 (17%) have at least five ESTs for e as well as i transcripts. Likewise, in 85 cases (77%) the minor splice form is confirmed by more than 1% of the ESTs that are spliced at the tandem donor, and in 49 cases (45%) this fraction is at least 5%. Thus, although we cannot exclude that some confirmations of GYNGYN tandem donors represent rare errors of the splice machinery, the majority seems to comprise real alternative splice events.

A or G is strongly preferred at intron position +3 for standard donor sites GTN, while T and C have lower frequencies [23]. We classified the confirmed GTNGTN donors according to their pattern into three groups: GTRGTR (R = A or G); GTTGTR, GTRGTT or GTTGTT; and GTCGTN or GTNGTC. The GTRGTR pattern is clearly preferred as 86% (70 of 81) of the confirmed GTNGTN donors belong to this group. A smaller fraction has one or two T at the N-positions (8 of 81, 10%) and the third group is very rare, with only three cases. These findings indicate that the common splicing machinery is operating at these sites. For GTNGCN and GCNGTN donors, we found very similar results: 21 of 29 (72%) have R at both N-positions and two (7%) one T. In addition, we found the exceptional pattern GTAGCC six times (21%).

**Figure 2:**
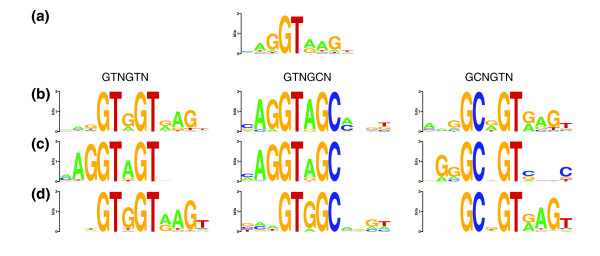
Sequence logos of the 12 nucleotide donor context (3 nucleotides upstream to 6 nucleotides downstream of the GYN). **(a) **Donors without a GYNGYN motif; **(b) **and the logo of the 12 nucleotide context (3 nucleotides upstream to 3 nucleotides downstream of the GYNGYN pattern) for GTNGTN, GTNGCN and GCNGTN donors classified into confirmed; **(c) **unconfirmed with annotated i donor; **(d) **unconfirmed with annotated e donor. Note that unconfirmed GTNGCNs with annotated e donor comprise only ten cases and unconfirmed GCNGTNs with annotated i donor only six cases. Sequence logos were generated with WebLogo [69].

Furthermore, we generated a sequence logo for the genomic context of confirmed tandems, unconfirmed GYNGYNs where either the e or i donor is confirmed, and donor sites without a GYNGYN motif (Figure 2). The three nucleotides up- and downstream of confirmed tandem donors are non-randomly distributed (Figure 2b), consistent with the observation that both donor sites are alternatively used in the splice process. In contrast, either the upstream or downstream side of unconfirmed GYNGYNs is more randomly distributed. The higher conservation of the AG upstream of the unconfirmed GTNGTN and GTNGCN motifs with annotated i donor (Figure 2c) indicates that the non-consensus intronic sequence (compare Figure 2a) is compensated by a more stringent match to the exonic part of the donor consensus sequence.

In some cases it has been reported that single nucleotide polymorphisms (SNPs) in the vicinity of donor sites lead to a shift in the splice site [24-26]. To check if there is a general trend that confirmed GYNGYNs might be influenced by SNPs in their genomic flanks, thus giving rise to allele-specific splice forms [27], we selected all SNPs from dbSNP that are mapped to the 100 nucleotide context up- and downstream of these tandem donors. We found that 64 (58%) of the confirmed GYNGYNs do not have an annotated SNP in this 206 nucleotide region. As a control we randomly selected 500 unconfirmed GYNGYNs and found that 56% (279 of 500) do not have a SNP in this 206 nucleotide region. Thus, we conclude that most of the confirmed tandems are not associated with allele-specific splice forms.

### Experimental verification of alternative GYNGYN splicing

**Figure 3:**
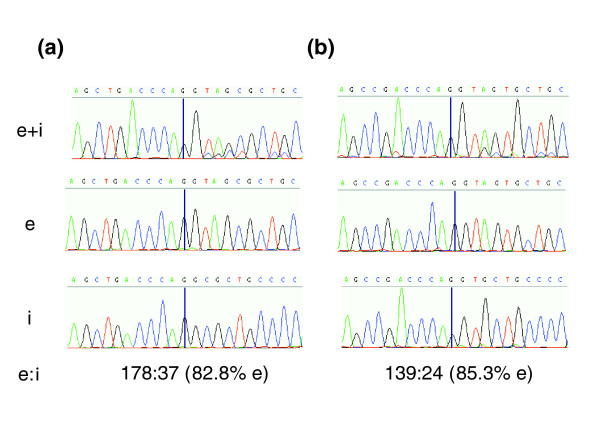
Alternative splicing at the tandem donor of exon 21 of **(a) **human *STAT3 *and **(b) **mouse *Stat3*. Electropherograms are shown for direct sequencing of RT-PCR amplicons (e+i) and sequencing of isolated clones representing e and i transcripts (e and i, respectively). The cursor is positioned on the nucleotide upstream of the conserved GTAGTT motif. Numbers and ratios of clones representing e and i transcripts are given for human and mouse kidney (e:i).

To further support the EST-derived confirmation of alternative splice events at tandem donor sites, we performed RT-PCR in several human tissues. We selected eight genes with confirmed GYNGYNs having at least three ESTs for e and i transcripts (Table 2, Figure 3a). We directly sequenced the RT-PCR products and inspected the sequencing traces for overlapping trace signals after the exon-exon junctions (Figure 3, e+i). This approach is based on control experiments showing that minor splice forms with a frequency down to 10% of the total transcripts can be clearly detected by direct sequencing (Additional data file 5). For seven of these eight GYNGYNs, we found e and i transcripts in all tissues where expression of the respective gene was observed. We detected no variation among the tissues, suggesting that these seven tandem donors are not regulated in a tissue-specific manner. Next, we analyzed the splicing at the tandem donor of *STAT3 *in leucocytes of six individuals and consistently observed both transcripts. This agrees with our *in silico *finding that tandem donor splicing in general does not depend on specific genotypes and further excludes the possibility that a peculiarity of the spliceosome or its components is the reason for the two splice forms.

### Differences in U1 snRNA binding for confirmed and unconfirmed GYNGYN donors

The U1 snRNA determines the donor site by base pairing with the mRNA [20]. To define the strength of a donor site, we calculated: the average free energy of U1 snRNA binding; the average number of base pairs between donor sites and U1 snRNA [28]; and the maximum entropy scores [29]. In general, the e donor of confirmed GTNGTNs has a higher strength compared to the i donor (Additional data file 2). In agreement with that, the e donor is annotated in 73% (59 of 81) of the confirmed GTNGTN donors in RefSeq. Furthermore, the e donor is represented by an average of 233 ESTs, which is about tenfold higher than the average of 24 ESTs for the i donor. These findings can be explained with a stronger consensus sequence downstream of a standard GT donor compared to the three upstream positions (Figure 2a). For GTNGCN and GCNGTN tandems, we have to distinguish between the GT and GC donor site since GT is stronger than GC (Additional data file 2). Consistently, of the 29 confirmed GTNGCN and GCNGTN tandems, the GT donor is annotated in 23 cases (79%) in RefSeq and the splicing at the GT donor is represented by an average of 116 ESTs compared to the average of four ESTs for GC donors.

**Figure 4:**
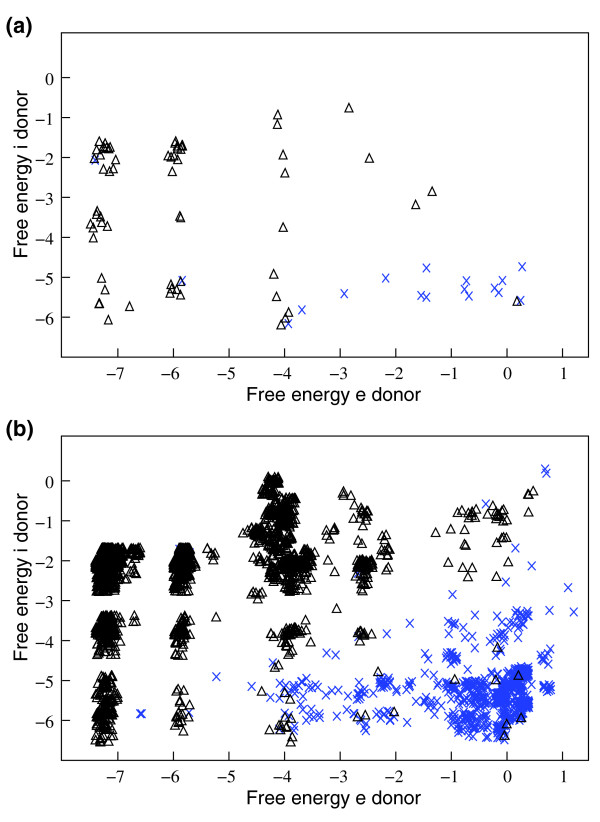
The free energy values in kcal/mol for e and i donor of human **(a) **confirmed GTNGTNs and **(b) **unconfirmed GTNGTNs. In (a) black triangles represent tandem donors with more ESTs for the e donor, and blue crosses tandem donors with more ESTs for the i donor. In (b) black triangles represent annotated e donors, and blue crosses annotated i donors. To better illustrate the distribution of the free energies, we added a random number between -0.1 and 0.1 to each value (necessary since many donor sites have the same 9 nucleotide context pattern).

Nevertheless, there are 17 of the 81 confirmed GTNGTN tandems with more ESTs for the i donor than the e donor. Therefore, we compared the free energy values and found that 15 of these 17 cases (88%) have a lower free energy for the i donor, thus allowing a more stable U1 binding (Figure 4a). Likewise, 56 of the remaining 64 confirmed GTNGTNs (88%) with more ESTs for the e donor have a lower free energy for the e donor. For GTNGCN and GCNGTN tandems, we found very similar results as in 90% (26 of 29) the donor with the lower free energy is represented by more ESTs. In agreement with other experimental and computational studies [30-32], the free energy of the U1 snRNA binding generally determines the donor that is used more frequently.

Since only a small fraction of all human tandem donors are confirmed, we searched for differences between confirmed and unconfirmed ones. Plotting the free energy values for e and i donors shows that most confirmed GTNGTNs are located at the left part (Figure 4a) while unconfirmed GTNGTNs can be separated by having a low free energy for either the e or the i donor (Figure 4b). Comparing the average free energies, we found that the e as well as the i donor of confirmed GYNGYNs is significantly stronger than the respective unannotated donor of unconfirmed GYNGYNs (Table 3, t-test, P-value < 0.00001). In contrast, the annotated donor of unconfirmed GYNGYNs is significantly stronger than the respective donor of confirmed GYNGYNs (Table 3, t-test, P-value < 0.00001). We repeated this analysis using the average number of base pairs and the maximum entropy scores to measure the strength of donor sites and found the same results (Table 3, t-test, all P-values < 0.00001). Dividing the confirmed tandem donors into GTNGTNs, GTNGCNs and GCNGTNs also leads to consistent results (Additional data file 2). Thus, unconfirmed tandem donors are characterized by a strong donor that successfully competes for U1 snRNA binding with the much weaker donor. The smaller difference between both donors for confirmed tandems probably allows U1 binding to both sites, leading to the observed splice variants.

We assumed that the strength of both donors might be a criterion to distinguish functional from non-functional tandem donors. To test this experimentally, we selected nine unconfirmed GTNGTNs with a low free energy for both donor sites for experimental verification. As for confirmed GTNGTNs, RT-PCR products were directly sequenced and the sequencing traces were inspected for overlapping sequences. For none of the nine candidates, we found evidence for alternative splicing at the tandem donor, suggesting that the majority of unconfirmed GTNGTNs is presumably not alternatively spliced. However, our direct sequencing approach does not exclude that the alternative transcript is expressed at a low frequency.

We conclude that: stable U1 binding is necessary but not sufficient for alternative tandem donor splicing; the currently confirmed GYNGYN represent a large fraction of all functional tandem donors; and, in contrast to NAGNAG acceptors [14], alternatively spliced GYNGYNs are not easily predictable.

### Confirmed tandem donors have over-represented motifs in their intron flanks

Since the free energy of U1 binding seems not to be the only discriminative criterion, we searched for other differences between confirmed and unconfirmed GTNGTNs. The regulation of alternative splicing often involves auxiliary exonic and intronic splice enhancer and silencer elements (abbreviated ESE, ESS, ISE, and ISS, respectively) that are bound by trans-acting RNA-binding proteins like serine/arginine rich (SR) proteins and hnRNPs [33-36]. Previous computational studies followed by experimental verification identified 238 hexamers as ESEs [37], 2,060 octamers as ESEs and 1,019 octamers as ESSs [38], and 133 hexamers as ISE motifs in the vicinity of donor sites [39]. We used these motifs to compare their average frequency between both groups. The 100 nucleotide exonic flanks of confirmed GTNGTNs are indistinguishable from unconfirmed ones when comparing the frequency of the 238 ESE hexamers (average of 10 ESEs per exon flank for both groups) and have a slight but not significantly higher frequency of ESE octamers (average 7.2 versus 6.5). The ESS frequency is slightly, but not significantly, lower for exon flanks of confirmed tandem donors (average 1.3 versus 2). However, we found a significantly higher frequency of ISE motifs in the 100 nucleotide intron flanks for confirmed GTNGTNs (average 10 versus 8, t-test: P = 0.0174). We repeated this analysis using a shorter exonic/intronic context (50 nt) and found consistent results (data not shown).

To find out if specific ISE hexamers are statistically over-represented, we used a resampling strategy. We randomly sampled 10,000 sets, each comprising 81 intron flanks from unconfirmed GTNGTNs. We estimated the P-value as the fraction of random sets with a higher frequency of a given ISE hexamer compared to the observed frequency in confirmed tandem donors. CGGGGT is the only one among the 133ISE motifs that is significantly over-represented in the vicinity of confirmed GTNGTN donors as all 10,000 random sets have a lower frequency (P < 1/10,000 × 133 = 0.0133 to correct for multiple testing). To find out if other sequence motifs are over-represented in the intron flanks of confirmed tandem donors, we repeated this procedure with tetramers. A word length of 4 nucleotides was chosen to account for the rather small data set. We only compared the 119 tetramers that occur at least with the expected frequency in the intron flanks of confirmed GTNGTNs. We found a significant overrepresentation for GGGT and CGGG (both have a higher frequency in only two random sets, P < 3/10,000 × 119 = 0.0357), while the tetramer GGGG has a corrected P-value slightly above the 0.05 threshold (higher frequency in five random sets, P < 0.0714). Since both GGGT and CGGG are substrings of the over-represented ISE CGGGGT, no new sequence motifs were found. The common feature of the over-represented sequence motifs is the G triplet. Interestingly, this motif occurs in 82 of the 133 ISEs [39] and is a known splice enhancer [40]. Since both splice sites of confirmed GTNGTNs are weaker compared to the annotated splice site of unconfirmed ones (Table 3), the G triplets might simply be associated with weak GTNGTNs. To exclude this possibility, we compared the average GGG frequency with unconfirmed GTNGTNs having a low U1 binding potential for both e and i donor (average free energy -3 kcal/mol for the e donor, -2.2 for the i donor) and still found an over-representation in the intron flanks of confirmed GTNGTNs (average 4.4 versus 2.6 G triplets per intron flank). Since this triplet was found to be more frequent in shorter introns [41], we divided our confirmed and unconfirmed datasets into short and long introns using 200 nucleotide as a cut-off. Consistently, the GGG is more frequent in the flanks of short as well as long introns with confirmed GTNGTNs (average 8.3 versus 4.4 G triplets per short intron, average 3.4 vs 2.7 per long intron). We also observed a noticeable higher ISE hexamer frequency in the intronic flanks of confirmed GTNGCN and GCNGTN tandems (average 12.7 versus 10.8, not significant), but only a slightly higher frequency of ESE hexamers and octamers in their exon flanks (data not shown). Specifically, G triplets are also more frequent in the intronic flanks of confirmed GTNGCN and GCNGTN donors compared to unconfirmed ones (average 5 versus 4.1). Thus, the occurrence of G triplets is another discriminating criterion between confirmed and unconfirmed tandem donors.

### Protein impact of alternative splicing at GYNGYN donors

Of the 81 confirmed GTNGTNs, 72 (89%) are located downstream of a coding exon; thus, alternative splicing at these sites results in 3 nucleotide indels into the coding sequence. The effect for the protein depends on the phase of the intron as well as the sequence of the GTNGTN and the upstream/downstream exon. In intron phase 0 (intron location between two codons) the GTN of the i donor is inserted/deleted and codes for a valine. In intron phase 1 and 2 (location between the first and second codon position, respectively), three different events are possible: indel of a single amino acid; exchange of a dipeptide and a different amino acid; and indel of a stop codon. Of the 72 GTNGTNs, 37 (51%) are located in phase 0, thus a valine indel is the most frequent event at the protein level. Of the 28 (39%) GTNGTNs in phase 1, 18 result in single amino acid events (14 times glycine, 2 times arginine, 2 times serine), 8 exchange a dipeptide and an unrelated amino acid and in two cases splicing at the i donor creates a stop codon. The 7 (10%) confirmed GTNGTNs in phase 2 are interesting since they either result in indels of rare amino acids (three times tryptophan, one cysteine, one tyrosine) or insert/delete a stop codon in two cases.

Thus, alternative splicing at four tandem donors has a more drastic impact on the proteins by the indel of a stop codon (phase 1: *FAM65A*, NM_024519, exon 21; *BRSK2*, NM_003957, exon 19; phase 2: *ABC1*, NM_022070, exon 8; *KLHL5*, NM_001007075, exon 3). In two cases (*KLHL5*, *ABC1*) the splice form with the premature stop codon is a clear candidate for nonsense-mediated mRNA decay [42], which potentially results in a down-regulation of the protein level. For the other two cases, the tandem donor affects the last intron of the transcript, thus the stop codon-containing splice variant should be translated into a protein with a shortened carboxyl terminus (*FAM65A *33 residue deletion at the carboxyl terminus, *BRSK2 *7 residue deletion).

The protein impact of the 28 GTNGCN and GCNGTN tandems that are located within the CDS comprise 22 single amino acid indels (intron phase 0: alanine, valine; phase 1: arginine, glycine, serine; phase 2: arginine, glutamine, leucine) and 6 dipeptide exchanges (all in phase 1). Compared to the protein events for GTNGTNs, the indel of alanine, glutamine and leucine is only observed for tandems with a GC donor.

Next, we compared the frequency of single amino acid events in phase 1 and 2 for confirmed and as a control for unconfirmed GTNGTNs. While only 42% (495 of 1,180) unconfirmed tandem donors in phase 1 result in a single residue indel, this percentage is significantly higher for confirmed tandems (64%, 18 of 28, Fisher's exact test: P = 0.02). The small number of phase 2 tandems does not allow a significant result, although the same trend is visible (100%, 5 of 5 confirmed tandems; 76%, 431 of 566 unconfirmed tandems; leaving stop codon events out). These findings argue for a preference to insert/delete only single amino acids, presumably because this is a less dramatic event compared to dipeptide exchanges. However, we cannot exclude the possibility that this result is an indirect consequence of a sequence bias of the GTNGTN motif and its context for confirmed tandems that primarily aims at a more stable U1 snRNA binding. For GTNGCN and GCNGTN tandems, only phase 2 donors result in a high percentage of single amino acid indels (100%, 5 of 5 confirmed; 81%, 781 of 962 unconfirmed) while phase 1 events show no bias (40%, 4 of 10 confirmed; 48%, 524 of 1103 unconfirmed).

### Tandem donors in seven other species

Further, we asked whether alternative splicing at GYNGYN donors is limited to humans or a general phenomenon. Therefore, we extended our analysis to the RefSeq transcripts of mouse (*Mus musculus*), rat (*Rattus norvegicus*), chicken (*Gallus gallus*), zebrafish (*Danio rerio*), fruitfly (*Drosophila melanogaster*), and nematode (*Caenorhabditis elegans*). The percentage of GTNGTN motifs in all donor sites is quite similar in all species and ranges between 2.1% and 3.4% (Table 4). For mouse, rat, zebrafish, fruitfly, and nematode, evidence for alternative splicing is found for 0.8% to 1.5% of all GTNGTN donors (Additional data file 3). The minor splice form is represented by at least 5% of the ESTs that are spliced at the tandem donor for most confirmed GTNGTNs (47% for mouse, 83% for rat, 60% for zebrafish, 90% for fruitfly, 92% for nematode), making splicing errors unlikely. Furthermore, 53% (26 of 49) of the mouse tandem donors have at least two ESTs for both splice forms, while this percentage drops for the other species due to the lower EST number (25% for rat, 60% for zebrafish, 42% for fruitfly, 31% for nematode). For chicken, we found only one confirmed tandem donor (0.2% of all GTNGTNs). Whether this lower percentage points at an exception for chicken is difficult to assess and deserves further research as the number of RefSeq transcripts and ESTs is rather limited. Next, we searched for GTNGCN and GCNGTN donors in those six species and, as for GTNGTNs, we found a comparable percentage of tandem donors and confirmed ones in all species (Table 4, Additional data file 3). Finally, we searched tandem donors in the plant *Arabidopsis thaliana *using the CDS annotation from GenBank and detected 36 confirmed GTNGTNs and 8 confirmed GCNGTNs/GTNGCNs (Table 4, Additional data file 3). Thus, all investigated species are able to produce e and i transcripts at tandem donors by alternative splicing and this phenomenon is not restricted to humans.

As in humans, the preferred motif for confirmed GTNGTNs is GTRGTR in all species except for *C. elegans *and *A. thaliana *where a higher fraction of GTNGTNs has one or two Ts at the N-positions (Table 5). The corresponding nucleotide in U1 snRNAs is a T that is post-transcriptionally modified to a pseudouridine (ψ), thus allowing base pairings with A or G [43,44]. We could not find an U1 snRNA gene with a different nucleotide at this position in the *C. elegans *or *A. thaliana *genome. Thus, except for the possibility of non-canonical ψ-T base pairings that have been observed at position +3 [30], we currently have no other explanation for the higher percentage of GTT tandem donors in these two species.

### Conservation of exonic and intronic flanks in mouse

Having observed several alternative GTNGTN splice events in human and mouse, we found conservation of the GTNGTN motif for 53 (65.4%) of the 81 human confirmed GTNGTNs. To assess whether this percentage is high or not, we counted GTNGTN conservation for the 3,909 unconfirmed tandems (162 of the 4,071 have no orthologous locus in mouse) and found a very similar percentage of 65.5% (2,561 of 3,909). The fraction of tandem donors that have a completely identical GTNGTN pattern in mouse is also equal: 40 of 81 (49.4%) confirmed, 1,939 of 3,909 (49.6%) unconfirmed. Thus, there is no evidence for a general selection pressure to maintain confirmed tandem donors since the divergence of the human-mouse ancestor.

However, a considerable fraction (10 of 53 (19%)) of the conserved and confirmed human GTNGTNs is also confirmed in mouse. For example, the GTAGTT donor of intron 21 of *STAT3 *is conserved in mouse and both e and i transcripts are supported by mouse ESTs. As in humans, we performed RT-PCR in several mouse tissues to further support the EST-derived confirmation of alternative splice events (Figure 3b). We found experimental evidence for alternative splicing at the *Stat3 *tandem donor in all investigated tissues and observed a strikingly similar trace pattern in human and mouse (Figure 3, e+i). Accordingly, the ratio of e and i transcripts estimated by the EST/mRNA counts are virtually identical (e transcripts 57 of 74 (77%)) human ESTs vs 55 of 69 (79.7%) mouse ESTs). To accurately quantify the ratio of e and i transcripts in one selected tissue, we subcloned the RT-PCR product, sequenced individual clones and found a remarkable agreement in the transcript ratio: 82.8% of the human clones indicate splicing at the e donor, which is almost equal to 85.3% in the mouse (Figure 3, e:i). Interestingly, this tandem donor is conserved in several other mammals and the e:i ratio is very similar (9:2 ESTs for rat, 12:3 ESTs for cow, 9:1 ESTs for dog). This indicates that, in addition to the tandem donor, regulatory elements may be conserved.

**Figure 5:**
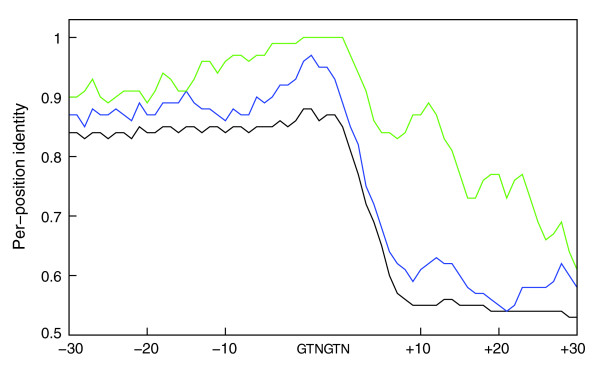
Per position identity values in human mouse alignments for the region 30 nucleotides up- and downstream of the GTNGTN motif. The black line represents unconfirmed human GTNGTN donors, the blue line confirmed human tandem donors with a conserved GTNGTN motif in mouse, and the green line conserved GTNGTNs that are confirmed in human and mouse. To avoid large variations due to the low case numbers, we calculated for each position the average of this and the three positions up- and downstream.

The intronic flanks of alternative exons are significantly more conserved in mouse compared to the flanks of constitutive exons, a fact which is presumably attributed to the force to maintain regulatory elements [45]. From the human-mouse genomic alignments, we calculated a per-position identity value for the region 30 nucleotides up- and downstream of the GTNGTNs. For a specific position, this value is the fraction of identical nucleotides in all alignments [45,46]. We calculated per-position identities for three groups: group 1, confirmed human tandem donors with a conserved GTNGTN motif in mouse; group 2, the subset of group 1 that is confirmed in human and mouse; and group 3, unconfirmed human tandems. Plotting these average values, it can be seen that group 1 and, in particular, group 2 have noticeably higher identities for both the exonic and intronic side compared to group 3 (Figure 5). The exonic identities for the 10 human and mouse confirmed and conserved tandem motifs exceed 90% for most positions, a feature that is also typical for alternative exons [47]. Furthermore, the GTNGTN pattern with 3 nucleotides up- and downstream is completely identical between both species for these 10 tandems and average identities of more than 80% are observed for the first 13 intronic positions.

## Discussion

We report the occurrence of alternative splice donor usage for GTNGTN, GTNGCN, and GCNGTN motifs in eight investigated eukaryotic species. Apart from our experimental verification of seven human and one mouse GYNGYN donors, several lines of evidence indicate that the majority of observed events is attributable to real alternative splicing. Firstly, numerous GTNGTNs are confirmed by multiple ESTs/mRNAs and for several of these events both e and i transcripts are deposited in the RefSeq database. Secondly, the existence of orthologous tandem donors that are confirmed in two or more species makes EST artifacts or database errors unlikely. Thirdly, these GTNGTN donors have a higher conservation of the exonic and intronic flanking regions, a situation that is typical for conserved alternative splice events [45,46,48,49]. Fourthly, all of the six investigated human individuals express e and i transcripts for *STAT3*, thus excluding the possibility of allele-specific instead of alternative splicing [27]. Finally, by manual examination of all human confirmed GYNGYNs, we excluded the existence of paralogs or processed pseudogenes that could mimic alternative splicing at a tandem donor.

We found that the percentage of donor sites with a GYNGYN motif as well as the percentage of tandem donors that are confirmed is very similar between the eight investigated species (tolerating some variation probably due to differences in the number of ESTs and mRNAs). Given the large evolutionary distance between *C. elegans*, *A. thaliana *and humans, it is likely that all species that have alternatively spliced genes are able to produce e and i transcripts at certain tandem donor sites. The detection of 44 alternatively spliced tandem donors in *A. thaliana *is consistent with the finding that alternative splicing in plants is not as rare as thought for a long time [50,51]. Previously, we have found that alternative NAGNAG splicing is widespread in human, mouse, and fruit fly but not in *C. elegans *[12]. To compare the numbers of GYNGYN donors and NAGNAG acceptors, we extended and updated our previous NAGNAG analyses [12,14] to the seven species having a RefSeq annotation in the UCSC Genome Browser (Table 6). In general, the percentage of confirmed NAGNAGs is one order of magnitude higher compared to GYNGYN donors. This can be explained by large differences in the mechanisms of donor and acceptor site recognition. While the acceptor AG is bound by U2AF35, the donor site is recognized by base pairing with the U1 snRNA. In contrast to the acceptor, the binding site of U1 comprises a larger range that is visible by the non-random nucleotide distribution for the last three exonic and first six intronic positions (Figure 2a). This imposes more sequence constraints on a tandem donor site and prevents the extensive use of potential e and i donors compared to potential E and I acceptors. Apart from human and mouse, the fruit fly has a relatively high percentage of confirmed NAGNAG sites, which is probably due to the higher percentage of tandem acceptors with the HAGHAG (H = A, C, or T) pattern that preferably allow alternative splicing. In contrast, a very low fraction of the NAGNAG acceptors of *C. elegans *is confirmed, which is particularly striking since *C. elegans *has the highest fraction of HAGHAG acceptors (Table 6). This rareness of alternative splice events at NAGNAG acceptors is not due to differences in the EST coverage as *C. elegans *has the similar percentage of confirmed tandem donors compared to the other species (Table 4). Therefore, it should be attributed to the unusual properties of the 3' intron ends of *C. elegans *that often lack consensus sequences for the branch point and the polypyrimidine tract [52].

Although only a fraction of the tandem donors is confirmed, we found features that distinguish confirmed from unconfirmed ones. Since the non-annotated donor of unconfirmed tandems does not allow a sufficiently stable binding to the U1 snRNA, the other donor is used exclusively in the splice process. For confirmed tandem donors, both sites allow a stable binding to U1 snRNA. However, in most of the confirmed cases one donor has a better strength and this results in its preferred usage as measured by the EST ratio between both transcripts. The second discriminative feature is the overabundance of G triplets in the intronic flanks of confirmed GTNGTNs, especially for introns shorter than 200 nt. This triplet is the core of many known ISE motifs [39,40] and was demonstrated to function in splice site definition [41]. Interestingly, in the human alpha-globin gene, GGG elements were shown to exert their effect by binding to the nucleotides 8-10 (5'-CCT-3') of the U1 snRNA [40]. We have searched for over-represented tetramers and found a significantly higher frequency of CGGG and GGGT. Strikingly, the nucleotides 7-11 of U1 snRNA are 5'-ACCTG-3'. The CGGG as well as the GGGT motifs are complementary to this part of U1; thus, it is tempting to speculate that these motifs bind to U1 snRNA with four instead of three base pairs. Since CGGG and GGGT are more frequent in the intronic flanks of confirmed tandem donors, they may be involved in alternative splicing at these donor sites. If U1 snRNA is a critical factor, we do not expect much variation in splicing between tissues since U1 is ubiquitously expressed in high amounts. Consistent with this notion, the seven experimentally investigated tandem donors exhibit similar e:i transcript ratios in all tissues.

Most confirmed GYNGYNs have a low free energy of U1 snRNA binding to both the e and i donor, suggesting that the U1 snRNA can stably bind to both sites. However, there are a few exceptions where one donor is much stronger than the other one in a confirmed tandem (Figure 4a). The mechanism of splicing at these sites remains unclear but there are several hypotheses that might guide future investigations. For example, it has been reported that U6 snRNA rather than U1 snRNA determines a donor site in the human *FGFR1 *gene [53]. Moreover, there is evidence that splicing can occur without U1 snRNA binding to the donor site [54,55]. Furthermore, other protein factors can influence the splice site choice and/or (de)stabilize U1 snRNA binding [56,57]. We believe that a further experimental investigation of confirmed tandem splice donors may help to elucidate further details of the splicing process.

Previously, we found that the impact of SNPs in NAGNAG acceptors on alternative splicing can be accurately predicted [14]. Therefore, it would be interesting to check if similar statements are possible for SNPs in GYNGYN donors. In principle, a SNP in close proximity to an unconfirmed GYNGYN donor might increase the base pairing capability to the U1 snRNA for the alternative donor, thus enabling alternative splicing. SNPs that affect a confirmed tandem donor might weaken U1 binding for one donor and result in the exclusive usage of the other. During the SNP mapping, we found two SNPs in the GTNGTN motif of human confirmed tandem donors. For exon 4 of *FAM3B *(NM_206964), the verified SNP rs417708 results in two alleles, GTGGTA and GCGGTA. For the C allele, the free energy of the i and e donor is -3.4 and -6.2 kcal/mol, respectively, while this value is more balanced for the T allele (-5.3 for i and -5.9 kcal/mol for the e donor). This agrees well with the prediction of the splice site analysis server [58,59]. Thus, it is likely that only the T allele produces two splice forms. The second SNP (rs11672749) is especially interesting, since it affects the tandem donor of exon 5 of the maternally imprinted *PEG3 *gene (NM_006210, GTGGTG and GTGGGG alleles). Both homozygous G genotypes and heterozygous genotypes with a maternally inactivated T allele will result in an exclusive splicing at the i donor.

Higher eukaryotes typically express multiple transcripts and proteins from a single gene. A prominent mechanism is alternative splicing as about 74% of the human multi-exon genes express more than one splice variant [4]. Protein isoforms can also be expressed from paralogous genes. Large gene families are observed to have a reduced frequency of alternative splicing, consistent with the idea that the variability of those gene products comes from the divergence of the gene copies [60]. While most research focused on large changes introduced by alternative splicing, it is becoming clear that there is a surprisingly high number of very similar protein isoforms. There are several mechanisms to introduce subtle protein changes. The most widespread type is alternative splicing at NAGNAG acceptors [12,15]. Furthermore, very similar mutually exclusive exons can lead to similar but functionally different proteins [61]. Here, we found that alternative splicing at GYNGYN donor sites occurs in all eight investigated species. Despite not as frequent as confirmed NAGNAG acceptors, the diverse protein changes further contribute to the plasticity of these proteomes. Confirmed tandem donors and acceptors are able to insert 12 of the 20 different amino acids by single amino acid events and the dipeptide exchanges are even more diverse. Further flexibility comes from the simultaneous use of a GYNGYN donor and a NAGNAG acceptor for one intron (Figure 1b). Such an example is intron 9 of *BRUNOL4 *(NM_020180), for which we found 14 e-E, 3 i-E and 6 e-I transcripts in dbEST that result in protein forms with a GPA, AA, or GP peptide, respectively.

Despite many GYNGYN donors, we found only a minority that allows alternative splicing. Nevertheless, among the human confirmed and evolutionary conserved tandem donors we found a significant fraction to be confirmed in other species. Moreover, the splicing pattern of the *STAT3 *GTNGTN donor is strikingly equal in human and mouse. In light of the discussion about functional versus non-functional alternative splicing [21,62], this is a strong indication that these alternative splicing events are not splicing noise. Consistently, such subtle changes by alternative splicing may result in functional differences for the two proteins. An arginine insertion between two zinc fingers results in a human glucocorticoid receptor isoform (NM_001018075, exon 3, GTAGTG) with an activity reduced to 48% [63,64]. Interestingly, this tandem donor is also conserved and confirmed in mouse. A similar subtle 6 nucleotide shift at a GTAAATGT donor of *ALDH18A1 *results in an isoform that is insensitive to ornithine inhibition [65]. Furthermore, there are at least four reported cases of functional differences by alternative NAGNAG splicing [15-18]. Thus, subtle alternative splice events are interesting candidates for further research, especially since several of them occur in known disease genes [14].

## Materials and methods

### Identification of GYNGYN donors and NAGNAG acceptors

We downloaded from the UCSC Genome Browser [66] the Human genome assembly (hg17, May 2004) as well as RefSeq annotation (refGene.txt.gz, November 2005). We discarded transcripts with an erroneous open reading frame or ambiguous characters in their sequence. If more than one entry with the same RefSeq ID exists, we selected the transcript with the highest number of exons. From the transcripts, we extracted a list of unique genomic positions of donor sites. We checked the presence of a GT/GC dinucleotide at the annotated donor position and 3 nucleotides up- or downstream. We repeated this procedure for the other six eukaryotes having a RefSeq annotation in the UCSC Genome Browser. The respective genome assemblies are mm6 (March 2005) for *M. musculus*, rn3 (June 2003) for *R. norvegicus*, galGal2 (February 2004) for *G. gallus*, danRer2 (June 2004) for *D. rerio*, dm2 (April 2004) for *D. melanogaster*, and ce2 (March 2004) for *C. elegans*. For *A. thaliana*, we used the genome assembly (NCBI, build 5.0) to build a list of donor sites according to the CDS feature annotations in GenBank format and screened this list for donor sites with a GYNGYN sequence pattern.

For checking if a GYNGYN donor is confirmed (at least one EST/mRNA for the e as well as i transcript), we compiled two search strings: 30 nucleotides from the upstream exon and 30 nucleotides from the downstream exon for the i transcript; and 30 nucleotides from the upstream exon, the GYN of the i donor and 30 nucleotides from the downstream exon for the e transcript. Then, we used BLAST against all ESTs and mRNAs for the respective species, allowing at most one mismatch or one gap but demanding exact identity for the region 27-33 for i-transcripts and 27-36 for e-transcripts. The ESTs were downloaded from the UCSC Genome Browser (est.fa.gz, November 2005). mRNAs were downloaded from GenBank at the same date as the ESTs. For acceptor sites with a NAGNAG pattern, we repeated the analysis using analogous procedures and the same data described above.

### Conservation analysis in mouse

Human-mouse genomic alignments (hg17-mm6) were downloaded from the UCSC Genome Browser (vsMm6/axtNet, March 2005). We used the genomic position of human and mouse donor sites to select the respective alignment chain. From the alignments, we determined whether a human GTNGTN donor is conserved (there is also a GTNGTN motif in mouse) or completely identical. For the per-position identity computation, we considered the alignment part up to 100 positions upstream and downstream. For each position, we counted how often there is identity between human and mouse (Nid), and how often there is a mismatch or gap (Nmm). The per-position identity value is Nid/(Nid+Nmm). Alignment positions with an 'N' aligned to a nucleotide were ignored.

To find tandem donors that are orthologous and confirmed in human and mouse, we used BLAST with the human-confirmed search strings against the search strings of the mouse-confirmed GTNGTNs. Furthermore, we used BLAST with the human-confirmed search strings against the mouse ESTs and mRNAs. Using the UCSC and Ensembl genome browser, we manually checked each hit with an E-value of less than 1e-3 for being alternatively spliced in both species and for having a true orthologous relationship.

### Strength of a donor site

We extracted a 9 nucleotide genomic context (3 nucleotides upstream to 3 nucleotides downstream of the GYN) for the e and i donor of confirmed and unconfirmed GYNGYNs. The free energy and number of base pairs with the U1 snRNA were computed according to [28] with the Splice-site Analyzer tool [67]. The score according to the maximum entropy model [29] was computed using MaxEntScan [68].

### Motif search

We extracted the genomic sequence 100 nucleotides upstream (exonic) and 100 nucleotides downstream (intronic) of GTNGTN donor motifs. To identify over-represented ISE hexamer motifs, we used a resampling procedure to estimate the P-value for a higher frequency in the intronic flanks of confirmed GTNGTNs. To this end we randomly sampled 10,000 sets of 81 intronic flanks of unconfirmed GTNGTNs and computed the frequency for each of the 133 ISE motifs in the 10,000 random sets. The P-value for one ISE is the fraction of random sets with a higher frequency compared to the observed frequency for confirmed GTNGTNs. To correct for multiple testing, each P-value is multiplied by 133. For the general search for over-represented motifs, we decided to use tetramers (word length 4 nt) instead of hexamers since the dataset of the confirmed tandems is rather small. Since we were searching for over-represented motifs in the intronic flanks of confirmed GTNGTNs, we expected that such motifs occur at least with the expected frequency under a null model and with a significant higher frequency compared to the flanks of unconfirmed GTNGTNs. There are 97 overlapping tetramers in a 100 nucleotide sequence, thus we analyzed a total of 81 × 97 = 7,857 tetramer occurrences. For complete random sequences, each tetramer should occur 7857/256 = 30.7 times. Since intron sequences are not random, we found a total of 119 tetramers that occur 30 times or more in the flanks of confirmed GTNGTNs. For these 119 tetramers, we repeated the procedure described above but multiplied the P-value by 119.

### Experimental verification of alternative splicing at tandem donors

Eight genes with multiple EST evidence for alternative splicing at a tandem donor were analyzed by RT-PCR in different tissues by using cDNA from multiple tissue cDNA panels (BD Clontech Germany, Heidelberg, Germany) as PCR templates. Primers were designed for the exons flanking the tandem donor with distances to these donors that allow reliable amplification and sequencing. PCR was performed in a total volume of 25 μl using ReadyToGo PCR beads (GE Healthcare Europe, Munich, Germany) with 5 pmoles of each primer and 1 μl of cDNA. Cycling conditions were 94°C for 30 s followed by 35 cycles with 94°C for 20 s, 57°C for 30 s and 72°C for 30 s, followed by a final extension at 72°C for 10 minutes. Amplified fragments were precipitated with ethanol and ammonium acetate, washed with ethanol and sequenced using DyeTerminator chemistry (Applied Biosystems, Foster city, USA) and the respective PCR primers on a 3730 xl DNA Analyzer (Applied Biosystems). Genes and their primer sequences were: *TOM1 *(AGTTTGACATGTTTGCGCTG, GCAGCCTTAACACCAGAGGA); *STAT3 *(GCCATCTTGAGCACTAAGCC, GGTTCAGCACCTTCACCATT); *ANAPC4 *(AGATGCTGCAGGAATCGAAG, CTGGCTTTTGCAAACACTGA); *RBM10 *(AGGCTGGATCAGCAGACACT, TCCCTCTTAGAACCCTTGGC); *ANGPT1 *(ACAAGGAAGAGTTGGACACC, GGGATTTCCAAAACCCATTT); *SEMA5B *(AGCACGTCCTGTGGCATC, GTCCTCGTCTCGGTCCTTCT); *CXorf44 *(GAGGGCAGGACTATGGGAG, AAATACTTCTCCTTCATAGCGGA); and *LTBP1 *(GGACCTGTATTTGTCAAGCCA, TAATGCAGTGTCCTGCTCCA). In addition, Stat3 of *M. musculus *was analyzed in the respective Clontech mouse tissue panel by amplifying and sequencing the homologous region with the oligonucleotides GCCATCCTAAGCACAAAGCC and GGCTCAGCACCTTCACCGTT. All sequence traces have been deposited in the NCBI Trace Archive (TIs human: 1166719658-1166720385; mouse; 1166879453-1166879628). To estimate the relative amounts of e and i transcripts of human and mouse *STAT3*, we cloned the respective amplicons into pCR2.1-TOPO (Invitrogen, Karlsruhe, Germany) and propagated the clones in *Escherichia coli *TOP10 cells. Plasmids were isolated from several isolated clones and their inserts were sequenced using universal M13 primers.

The same strategy was applied to a set of genes with unconfirmed tandem splice donors. Genes and their primers were: *A2ML1 *(NM_144670, exon 17, ACTTTCCTCAGCCCCTCATT, AGTGCAGAAACTCATCGCCT); *TMEM63C *(NM_020431, exon 1, GTGCTGAGGACGCAAATCA, CATCTCCAAGGAAGTCTCCG); *RNPC3 *(NM_017619, exon 13, ACCGGGTGAACCAAACTGTA, AGCTGTTACGCACAGTTCCA); *GOLGA3 *(NM_005895, exon 5, CACCCCCTATATGGTCAACG, CACGACTGCTTCAGGGTGT); *ART5 *(NM_053017, exon 2, GCCCCTATACAGGCCTTCTC, ATTGCAACACCGTTCAATCA); *KIAA1853 *(NM_194286, exon 8, CCCTCAAGCTGTGAGAGCAG, TGGTGAAGGAGTTCCCTGAA); *K5B *(NM_173352, exon 5, ACAACAACCGCTACCTGGAC, AATCTCCACATCCAGGGAAA); *SAMD4B *(NM_018028, exon 15, AACAGCATGCCCAGTCAGA, CTCAGCAGAGATCCCTCGAC); and *LOC221711 *(NM_194299, exon 9, TTCAGATGTTGGATTCCTTCC, TTTTTCATCCGCTGGTTTTC).

### Data availability

To facilitate further experimental and computational studies of tandem splice sites, we recently developed a database, TassDB [70], which provides large collections of GYNGYN donors and NAGNAG acceptors of eight species.

## Additional data files

The following additional data are available with the online version of this paper. Additional data file 1 is an Excel spreadsheet containing data on all human confirmed GYNGYN splice donor sites identified in this study. Additional data file 2 is an Excel spreadsheet providing data about the strength of e and i human GYNGYN donors. Additional data file 3 is an Excel spreadsheet listing all confirmed GYNGYN donors for seven species. Additional data file 4 is an Excel spreadsheet presenting information about selected sequence traces that exemplify the experimental verification of GYNGYN donors. Additional data file 5 is a Word file describing the control experiments for detecting minor splice forms by direct sequencing of RT-PCR products.

## Supplementary Material

Additional data file 1 Data for all human confirmed GYNGYN splice donor sites identified in this study Click here for file

Additional data file 2 Data on the strength of e and i human GYNGYN donors Click here for file

Additional data file 3 Confirmed GYNGYN donors for seven species Click here for file

Additional data file 4 Selected sequence traces that exemplify the experimental verification of GYNGYN donors Click here for file

Additional data file 5 Control experiments for detecting minor splice forms by direct sequencing of RT-PCR products Click here for file

## Figures and Tables

**Table 1:** Human tandem donor sites divided into the four different GYNGYN patterns

Splice donor pattern	Number and % of tandem donors*		Number and % of confirmed donors	
GTNGTN	4,152	2.51%	81	1.95%
GTNGCN	856	0.52%	14	1.64%
GCNGTN	3,510	2.12%	15	0.43%
GCNGCN	32	0.02%	0	0.00%
GYNGYN	8,550	5.17%	110	1.29%

*Percent of all 165,295 annotated donor sites

**Table 2:** Experimental verification of human GYNGYN donors

Gene symbol	RefSeq ID	Upstream exon	Annotated donor	Pattern	Transcripts found*
*ANAPC4*	NM_013367	18	i	GTAGTA	e < i
*ANGPT1*	NM_001146	4	e	GTGGTA	e > i
*SEMA5B*	NM_001031702^†^	16	e	GTGGTG	e > i
*RBM10*	NM_005676^†^	10	e	GTGGTG	e < i
*TOM1*	NM_005488	14	i	GTAGTA	e < i
*STAT3*	NM_003150^†^	21	i	GTAGTT	e > i
*CXorf44*	NM_138362	2	e	GTAGCA	e < i
*LTBP1*	NM_206943	14	e	GTAGCC	e

*e < i means higher expression of i transcripts; e > i means higher expression of e transcripts; e means only e transcripts found. All sequence traces are deposited in the NCBI Trace Archive (Additional data file 4). ^†^The alternative donor is annotated in another RefSeq transcript.

**Table 3:** Characteristics of U1 snRNA binding to human confirmed and unconfirmed GYNGYN donors

	Average					
	
	Free energy (kcal/mol)*		Number of base pairs*		Maximum entropy score^†^	
	i	e	i	e	i	e
Unconfirmed, e annotated	-1.92	-5.46	3.12	6.69	-18.13	7.95
Unconfirmed, i annotated	-5.01	-0.24	6.49	3.30	4.63	-16.02
Confirmed	-3.68	-4.25	4.84	6.09	-5.88	1.65

*Computed with the Splice-site Analyzer tool [67]. ^†^Computed with MaxEntScan [68].

**Table 4:** GTNGTN, GTNGCN and GCNGTN donors in eight investigated species

Species	Number of donors*	GTNGTN				GTNGCN and GCNGTN			
		
		Observed		Confirmed		Observed		Confirmed	
*H. sapiens*	165,295	4,152	2.51%^†^	81	1.95%^‡^	4,398	2.66%^§^	29	0.66%^¶^
*M. musculus*	125,332	3,188	2.54%	49	1.54%	3,237	2.58%	12	0.37%
*R. norvegicus*	53,631	1,424	2.66%	12	0.84%	1,440	2.69%	3	0.21%
*G. gallus*	19,793	554	2.80%	1	0.18%	553	2.79%	2	0.36%
*D. rerio*	29,091	619	2.13%	5	0.81%	699	2.40%	1	0.14%
*D. melanogaster*	40,811	1,274	3.12%	19	1.49%	1,906	4.67%	5	0.26%
*C. elegans*	92,938	3,195	3.44%	26	0.81%	2,838	3.05%	1	0.04%
*A. thaliana*	112,684	3,541	3.14%	36	1.02%	2,091	1.86%	8	0.38%

*Total number of all unique donor sites annotated in RefSeq transcripts; for *A. thaliana *total number of unique donor sites based on the CDS feature annotation of GenBank. ^†^Number of observed GTNGTNs/number of all donors. ^‡^Number of confirmed GTNGTNs/number of observed GTNGTNs. ^§^Number of observed GTNGCNs and GCNGTNs/number of all donors. ^¶^Number of confirmed GTNGCNs and GCNGTNs/number of observed GTNGCNs and GCNGTNs.

**Table 5:** Number of confirmed GTNGTN donors divided into three groups according to their motif

	GTRGTR		GTRGTT + GTTGTR + GTTGTT		GTCGTN + GTNGTC	
*H. sapiens*	70	86%	8	10%	3	4%
*M. musculus*	44	90%	4	8%	1	2%
*R. norvegicus*	10	83%	2	17%	0	0%
*G. gallus*	0	0%	1*	100%	0	0%
*D. rerio*	5*	100%	0	0%	0	0%
*D. melanogaster*	14	74%	5	26%	0	0%
*C. elegans*	11	42%	15	58%	0	0%
*A. thaliana*	16	44.5%	16	44.5%	4	11%

*Case number too low to draw any conclusion.

**Table 6:** NAGNAG acceptors for seven species

Species	Number of acceptors*	NAGNAG acceptor		HAGHAG acceptor		Confirmed NAGNAG acceptor		Confirmed HAGHAG acceptor	
*H. sapiens*	164,841	9,465	5.7%^†^	3,530	37.3%^‡^	1,511	16%^§^	1,373	90.9%^¶^
*M. musculus*	125,233	7,116	5.7%	2,662	37.4%	1,087	15.3%	1,022	94.0%
*R. norvegicus*	53,598	3,080	5.7%	1,098	35.6%	215	7.0%	202	94.0%
*G. gallus*	19,794	1,069	5.4%	401	37.5%	97	9.1%	92	94.8%
*D. rerio*	29,067	1,540	5.3%	484	31.4%	132	8.6%	118	89.4%
*D. melanogaster*	39,441	1,584	4.0%	859	54.2%	177	11.2%	170	96.0%
*C. elegans*	92,867	4,184	4.5%	2,637	63.0%	33	0.8%	33	100.0%

H stands for A, C, or T. *Total number of all unique acceptor sites annotated in RefSeq transcripts. ^†^Number of NAGNAG acceptors/number of all acceptors. ^‡^Number of HAGHAG acceptors/number of NAGNAG acceptors. ^§^number of confirmed NAGNAG acceptors/number of NAGNAG acceptors. ^¶^Number of confirmed HAGHAG acceptors/number of confirmed NAGNAG acceptors.
