# Supplementary material for: Phylogenetically widespread alternative splicing at unusual GYNGYN donors
Source: Genome Biol. 2006 Jul 25;7(7):R65. doi: 10.1186/gb-2006-7-7-r65 (PMC1779574; doi:10.1186/gb-2006-7-7-r65)
Supplement: Additional data file 5 — Control experiments for detecting minor splice forms by direct sequencing of RT-PCR products [file gb-2006-7-7-r65-S5.doc]

**Supplementary Text**

**Control experiments for detecting minor splice forms by direct sequencing of RT-PCR products**

Two splice forms of human *CARD15* (skipping of exon 3, GenBank AY187243 and AY187246) were amplified by RT-PCR and subsequently cloned using primers CCTGGCATGGGAGCGG and CAGCAAGTGCAGCCGCTG and cDNA from leukocytes. PCR and cloning was performed as described in the Methods section of the manuscript. Two plasmids representing the alternative splice forms were identified by sequencing and used to prepare mixtures, mimicking in RT-PCR products the fraction of the minor splice form in the range from 5% to 50%. These mixtures were reamplified by PCR and directly sequenced (**Figure**).

These control experiments demonstrate that minor splice forms with a frequency down to 10% of the total transcripts can be clearly detected by direct sequencing of RT-PCR products. We demand (i) that - in respect to the sequencing direction - the traces show overlapping signals only after the exon-exon junction and (ii) that the overlapping signals represent exactly the superposition of the two splice forms we expect.

**A**

**B**

**C**


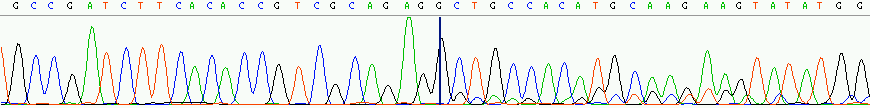

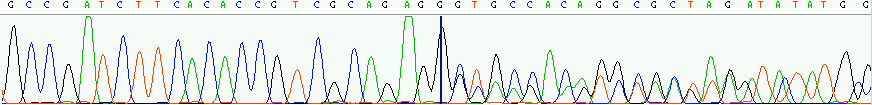

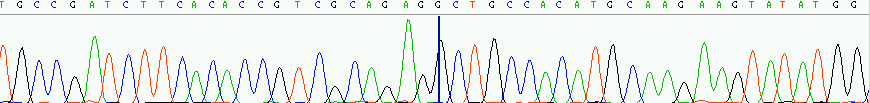


**Figure** Sequence traces obtained by direct sequencing of PCR products derived from mixtures of two plasmid clones representing alternative splice forms of human *CARD15* exon 3 skipping. Ratios of splice form with skipped *vs.* included exon: (**A**) 100:0, (**B**) 90:10 and (**C**) 70:30.

The cursor is positioned on the last nucleotide of exon 2. For (B) and (C) overlapping trace signals resulting from exon 3 (GCAAGAAGG...) and exon 4 (CTGCCACATG...) are clearly visible. Note that there is no overlap at position 6 downstream of the cursor since the sixth nucleotide of exon 3 and exon 4 is an adenosine. As expected, the trace signal of the major splice form (skipping of exon 3) is stronger.
